# Concurrent transcriptional profiling of *Dirofilaria immitis* and its *Wolbachia* endosymbiont throughout the nematode life cycle reveals coordinated gene expression

**DOI:** 10.1186/1471-2164-15-1041

**Published:** 2014-11-29

**Authors:** Ashley N Luck, Christopher C Evans, Molly D Riggs, Jeremy M Foster, Andrew R Moorhead, Barton E Slatko, Michelle L Michalski

**Affiliations:** New England Biolabs, Inc., Genome Biology Division, 240 County Road, Ipswich, MA 01938 USA; Department of Infectious Diseases, College Veterinary Medicine, University of Georgia, 501 D. W. Brooks Drive, Athens, GA 30602 USA; Department of Biology and Microbiology, University of Wisconsin Oshkosh, Oshkosh, WI 54901 USA

**Keywords:** Nematode, Filaria, Transcriptomics, Endosymbiosis, *Wolbachia*, RNA-seq

## Abstract

**Background:**

*Dirofilaria immitis*, or canine heartworm, is a filarial nematode parasite that infects dogs and other mammals worldwide. Current disease control relies on regular administration of anthelmintic preventives, however, relatively poor compliance and evidence of developing drug resistance could warrant alternative measures against *D. immitis* and related human filarial infections be taken. As with many other filarial nematodes, *D. immitis* contains *Wolbachia,* an obligate bacterial endosymbiont thought to be involved in providing certain critical metabolites to the nematode. Correlations between nematode and *Wolbachia* transcriptomes during development have not been examined. Therefore, we detailed the developmental transcriptome of both *D. immitis* and its *Wolbachia* (*w*Di) in order to gain a better understanding of parasite-endosymbiont interactions throughout the nematode life cycle.

**Results:**

Over 215 million single-end 50 bp reads were generated from total RNA from *D. immitis* adult males and females, microfilariae (mf) and third and fourth-stage larvae (L3 and L4). We critically evaluated the transcriptomes of the various life cycle stages to reveal sex-biased transcriptional patterns, as well as transcriptional differences between larval stages that may be involved in larval maturation. Hierarchical clustering revealed both *D. immitis* and *w*Di transcriptional activity in the L3 stage is clearly distinct from other life cycle stages. Interestingly, a large proportion of both *D. immitis* and *w*Di genes display microfilarial-biased transcriptional patterns. Concurrent transcriptome sequencing identified potential molecular interactions between parasite and endosymbiont that are more prominent during certain life cycle stages. In support of metabolite provisioning between filarial nematodes and *Wolbachia*, the synthesis of the critical metabolite, heme, by *w*Di appears to be synchronized in a stage-specific manner (mf-specific) with the production of heme-binding proteins in *D. immitis*.

**Conclusions:**

Our integrated transcriptomic study has highlighted interesting correlations between *Wolbachia* and *D. immitis* transcription throughout the life cycle and provided a resource that may be used for the development of novel intervention strategies, not only for the treatment and prevention of *D. immitis* infections, but of other closely related human parasites as well.

**Electronic supplementary material:**

The online version of this article (doi:10.1186/1471-2164-15-1041) contains supplementary material, which is available to authorized users.

## Background

*Dirofilaria immitis*, the causative agent of canine heartworm disease, is a parasitic filarial nematode evolutionarily related to those responsible for human parasitic diseases such as lymphatic and cutaneous filariases. Like other onchocercids, *D. immitis* requires an arthropod vector for transmission (in this case *Aedes*, *Anopheles* or *Culex* mosquitoes), as well as a mammalian host. Natural patent infections of *D. immitis* occur in canids, including domestic dogs, coyotes and wolves, but can also occur in other mammals such as cats, ferrets and even humans [1]. The life cycle of *D. immitis* follows that of other filarial nematodes in that infected insects, mosquitoes in this case, introduce third-stage larvae (L3) into the vertebrate host during a blood meal. The L3 larvae molt first into fourth-stage larvae (L4) and then adults within the vertebrate host. Adult males and females (AM and AF) residing in the pulmonary arteries of the mammalian host reproduce and give rise to microfilariae (mf), which are released into the blood [2]. Circulating mf are ingested by a mosquito during another blood meal and molt twice within the vector before becoming infective L3 larvae [3].

If left untreated, adult worms present in the pulmonary vessels of the dog result in prolonged physical damage and inflammation. As the inflammation resolves and fibrosis occurs, affected vessels become less elastic and signs of right-sided heart failure can ensue [4]. Worms can also become lodged in the heart and pulmonary vessels where they can block circulation. Typically, the severity of disease is related to the number of adult worms present, although other factors, such as dog size and response to infection, have been hypothesized to affect severity [1, 4]. Adult worms can be surgically removed with alligator foreceps, however, the currently recommended adulticidal treatment is the FDA-labeled adulticide melarsomine dihydrochloride, often used in conjunction with corticosteroids, aspirin and/or doxycycline [4]. While this drug is highly effective, treatment requires hospitalization with multiple intramuscular injections that can result in adverse side effects [4]. Furthermore, the relative cost of adulticidal treatment is typically greater than the cost of year-round monthly heartworm prevention. Adulticidal treatment is not recommended for use in cats, making prophylaxis the only option.

The development of heartworm disease is prevented by monthly administration of a macrocyclic lactone (*i.e*., ivermectin, selamectin, moxidectin or milbemycin oxime). These drugs kill susceptible L3s and L4s present in the animal. Elimination of these larval stages prevents the development of adult heartworms within the vertebrate host. As evidenced by estimated prevalence rates of up to 12.5% in the United States and much higher in other industrialized countries [5], there is, perhaps surprisingly, relatively poor compliance for domestic dogs. Factors shown to affect compliance include owner age, owner household income, and whether or not the pet is neutered [6]. Furthermore, resistance to current drugs is developing [7, 8]. The few isolates rigorously tested for genotypes conferring resistance suggest P-glycoproteins are involved in providing resistance [9–11], however, the exact molecular mechanisms remain unclear at this time. The increase in reports of loss of efficacy/prophylaxis highlights the need for vaccine development against *D. immitis* and other filarial nematodes.

As with many other filarial nematodes, *D. immitis* contains an obligate bacterial endosymbiont, *Wolbachia*, that is present in the lateral chords of both sexes as well as the oocytes within the female reproductive tract. The nature of the essentiality of the *Wolbachia-*nematode symbiosis remains unclear, but, based on genomic sequences, is thought to derive from metabolite provisioning between the nematode and bacterium [12]. Early anti-*Wolbachia* treatments (doxycycline) in dogs were based on earlier observations from laboratory animals (jirds) where antibiotic treatments reduced parasite loads [13–16]. More recently, combined doxycycline and ivermectin treatment in dogs has been shown to have both adulticidal [17] and microfilaricidal activity *in vivo*[18], further demonstrating the essential mutualism between *D. immitis* and *Wolbachia*.

Genomic DNA sequences of the *D. immitis* and *Wolbachia* of *D. immitis* (*w*Di) genomes were recently completed and published [19]. While extremely useful, genomic approaches alone cannot provide a detailed understanding of the symbiotic relationship between nematode and bacterium. Related “omics” studies (transcriptomics/proteomics) are necessary to supply detailed functional information, enable improved diagnostics and provide new drug and vaccine targets. In light of developing drug resistance, we initiated the first series of concurrent transcriptional profiling experiments throughout the nematode life cycle to understand the global concerted transcriptional activity of the *D. immitis* and *Wolbachia* genomes and provide further insights into the evolutionary biology of these parasites and their symbionts.

## Methods

### Parasites

The *D. immitis* used in this study was from a naturally infected dog maintained at the University of Georgia College of Veterinary Medicine. This research was approved by the University of Georgia Institutional Animal Care and Use Committee.

Microfilariae were collected in whole blood drawn from the jugular vein of the infected dog. Microfilariae concentration was determined by viewing Giemsa stained thick blood smears. The mf concentration throughout the study was approximately 12,000 mf/ml of blood (data not shown). L3 were obtained by feeding microfilaremic blood to *Aedes aegypti* mosquitoes (black-eyed Liverpool strain) using artificial blood feeders, as previously described [20]. Fourteen days after feeding, L3 were obtained by gently crushing the infected mosquitoes, rinsing them onto a 32 μm mesh sieve set in a petri dish and soaking them in warm Hanks’ balanced salt solution, with larvae settling to the bottom of the dish for collection.

L4 were cultured from day 14 L3 isolated using the aforementioned procedure. Larvae were washed in phosphate buffered saline (Boston BioProducts) and cultured in 24 well plates (Becton Dickinson). Culture media consisting of RPMI-1640 (Lonza), heat inactivated fetal bovine serum (Sigma-Aldrich), gentamicin (Sigma-Aldrich), and penicillin/streptomycin combination (Hyclone Laboratories, Inc.) was changed every two days for fourteen days. Fully cast cuticles were observed beginning day 3 and lasted through day 14 of culture when L4 larvae were collected and frozen in 250 μl of PBS. Microscopic examination of five randomly chosen worms confirmed that they were in the fourth larval stage (data not shown).

For collection of adult worms, the dog was necropsied and adult worms collected from the heart and pulmonary vessels. All worms were fast frozen in 1.5 mL graduated round bottom tubes (Eppendorf) at -80°C.

### Total RNA Isolation, Library Preparation and Sequencing

*D. immitis* samples were homogenized with ceramic beads in CK14 tubes using a Minilys homogenizer (Precellys) and total RNA was extracted by organic extraction using Trizol (Ambion). Samples were treated with DNase I (Ambion) before further Trizol extraction and final purification. The RNA integrity, purity and concentration of all samples were assessed using a Bioanalyzer 2100 (Agilent Technologies). In order to capture *w*Di transcripts, 100–125 ng of total RNA and the NEBNext^®^ mRNA Library Prep Master Mix Set for Illumina^®^ (Cat. # E6110, New England Biolabs) or the NEBNext^®^ Ultra RNA Library Prep Kit for Illumina^®^ (Cat. # E7530, New England Biolabs) were used to prepare the libraries according to the kit instructions. Library quality was assessed using a DNA high sensitivity chip on a Bioanalyzer 2100 prior to sequencing. Transcriptomic libraries (50 bp single end reads) were sequenced on a Genome Analyzer II× (Illumina). Two biological replicates (~18,500 or 33,200 mf/replicate, ~500 L3/replicate, 232 or 317 L4/replicate, 2 or 3 AF/replicate and 4 or 5 AM/replicate) were prepared and sequenced for each life cycle stage. No technical replicates were performed.

### Sequence Alignment and Differential Expression Analysis

All data were analyzed using a local instance of Galaxy [21–23]. Sequence reads from each sample were analyzed using the Tuxedo protocol [24]. Briefly, RNA-Seq reads were first assessed for quality based on quality scores per base using FastQC [25]. RNA-Seq reads from each sample were aligned to the *D. immitis* genome (version 2.2) [19] using TopHat (v. 1.4.1) [26], a mapper capable of identifying splicing variants and junctions within eukaryotic transcriptomes. Default parameters were used. Reads aligned using TopHat were first viewed using the Integrated Genomics Viewer (IGV) [27] before being assembled into transcripts using Cufflinks (v. 2.1.1, default parameters). Cufflinks assemblies from all samples were merged using Cuffmerge and Cuffdiff was employed for differential expression testing. In Cuffdiff, quartile normalization and multi-read correct options were used. The false discovery rate (FDR) was set to 0.01.

Similarly, the RNA-Seq reads from each sample were also mapped to the *w*Di genome (version 2.2) using Bowtie [28]. Reads aligned using Bowtie were assembled into transcripts using Cufflinks, then merged with Cuffmerge. Differential expression profiles were determined using Cuffdiff (v. 2.1.1). Default parameters for Cuffdiff were used except the minimum alignment count was set to 2 and FDR set to 0.01.

Hierarchical clustering analysis was performed using Cluster 3.0 [29]. Mapped reads from biological replicates (BAM output files from either TopHat or Bowtie) were first merged then assembled into transcripts using Cufflinks. Normalized FPKM (Fragments Per Kilobase of transcript per Million mapped reads) values were hierarchically clustered using Pearson’s uncentered correlation coefficient with a centroid linkage. Clustered data were depicted graphically (heatmap and dendrogram) using JavaTreeView [29]. GO terms were assigned to predicted *w*Di gene models using InterProScan (version 4) [30, 31]. Significantly enriched GO terms were identified using the web based Gene Ontology Enrichment Analysis Software Toolkit (GOEAST) [32] with the FDR set to 0.1.

## Results and discussion

### Transcriptome overview

In total, over 215 million single-end 50 bp reads were generated from total RNA from the *D. immitis* life cycle stages. Following the removal of low quality reads, approximately 55% of the sequenced reads mapped to the *D. immitis* reference genome (Table 1). Of the mappable reads, an average of 83.3% ±5.9% were uniquely mapped to the *D. immitis* genome, while the remaining ~16.7% of reads (likely ribosomal RNA) mapped to multiple locations within the genome. Interestingly, FPKM distribution and coverage of the 12,857 predicted gene models varied greatly among the different *D. immitis* life cycle stages (Additional file 1: Dataset S1; Additional file 2: Figure S1A, Table 1). Although many reads map to rRNA, ~80% of all *D. immitis* predicted gene models were expressed in every life cycle stage examined (Table 1).

**Table 1:** **Total number of reads sequenced and mapped to the** ***D*** *.* ***immitis*** **genome per** ***D*** *.* ***immitis*** **life cycle stage**

Sample	Million reads sequenced (Rep 1)	Million reads mapped (Rep 1)	Million reads sequenced (Rep 2)	Million reads mapped (Rep 2)	Million reads mapped (Reps Combined)	50th Percentile (FPKM)	75th Percentile (FPKM)	95th Percentile (FPKM)	Average coverage (FPKM)/Transcript	***D. immitis*** transcripts detected (%)**
**AM**	9.3	4.2	2.6	1.0	5.2	3.0	6.7	31.3	83.9	81
**AF**	14.8	7.2	1.4	0.5	7.7	7.3	14.9	53.4	92.0	93
**mf**	4.6*	1.6*	43.3	24.7	24.7	8.2	15.0	78.6	53.3	86
**L3**	20.7	8.7	27.3	12.2	20.9	0.8	2.1	15.3	45.4	81
**L4**	53.3	33.7	38.3	23.4	57.1	10.4	27.9	129.3	74.3	83

Each life stage replicate, as well as the combined total of mapped reads per life stage are listed. *Due to DNA contamination, this replicate was omitted from further analysis. **Based on number of transcripts expressed (FPKM > 0) per life cycle stage.

A pairwise comparison of replicates from each *D. immitis* life cycle stage can typically identify sampling or sequencing bias between the two biological replicates from each life cycle stage. As expected, most biological replicates are strikingly similar to one another (Additional file 2: Figure S1B-F). However, the *D. immitis* mf biological replicates displayed greater variation in gene expression than the other *D. immitis* life cycle stages (Additional file 2: Figure S1D). Closer interrogation revealed genomic DNA contamination of *D. immitis* mf biological replicate 1 (as indicated by reads mapping to intergenic regions in the *D. immitis* genome). Thus, for all further analysis, only data from *D. immitis* mf biological replicate 2 was utilized.

Because total RNA was used for library construction, the reads from the *D. immitis* life cycle stages (Table 1) were also mapped to the *Wolbachia* endosymbiont (*w*Di) genome. This approach was previously used to successfully sequence the transcriptome of *Wolbachia* from adult males and female gonads of the cattle parasite, *Onchocerca ochengi* (*w*Oo) [33]. As anticipated based on previous mixed transcriptomic studies [34], significantly fewer reads mapped to *w*Di genes (Table 2) than to *D. immitis* genes (Table 1) in each life cycle stage: on average only 0.7% of sequenced reads (ranging from 0.02% to 2.11% depending on the life cycle stage) mapped to *Wolbachia*. A relatively high number of *w*Di transcripts (above 90%) were detected by sequencing total RNA from *D. immitis* AF and mf, while fewer transcripts were detected in the AM, L3 and L4 *D. immitis* samples (~50-60% of transcripts, Table 2). The extremely low representation of *w*Di reads is expected in a mixed transcriptome [34], nonetheless, the low coverage of the L4 sample is surprising, especially since numerous reports suggest that *Wolbachia* numbers rapidly increase during the L4 stage in filarial nematodes [1, 35–37]. However, although *Wolbachia* read numbers in the L4 sample are low (especially compared to the relative nematode reads), the level of transcription (FPKM value) of certain genes in the L4 stages is relatively high compared to the other stages, suggesting that at least a certain subset of *Wolbachia* genes (and maybe more if the coverage were higher) are highly transcribed during the L4 stage. The variable and relatively low coverage of the *w*Di transcriptome in some life cycle stages required certain parameters be adjusted (the minimum alignment count to be lowered) in order to conduct differential expression significance testing. Despite an overwhelming amount of host transcripts and rRNA, using these parameters, 653 *w*Di genes were deemed differentially expressed (q < 0.01) between at least two of the five life cycle stages (Additional file 3: Table S1).

**Table 2:** **Total number of reads mapped to the** ***w*** **Di genome per** ***D*** *.* ***immitis*** **life cycle stage**

Sample	***w*** Di Mapped reads (50 bp)	***w*** Di Mapped bases	50th Percentile (FPKM)	75th Percentile (FPKM)	95th Percentile (FPKM)	Average coverage (FPKM)/Transcript	***w***Di transcripts detected (%)*
**AM**	115,048	5,752,400	91.0	215.4	1604.7	442.4	63
**AF**	340,598	17,029,900	171.4	375.4	2194.9	463.5	93
**mf**	110,894	5,544,700	1506.6	4047.7	22,418.4	5141.4	96
**L3**	26,673	1,333,650	0	106.6	769.1	287.3	48
**L4**	17,619	880,950	199.0	887.2	6480.2	1849.6	57

*Based on number of transcripts expressed (FPKM > 0) per life cycle stage.

### Stage-dependent transcriptional variation

**Figure 1:**
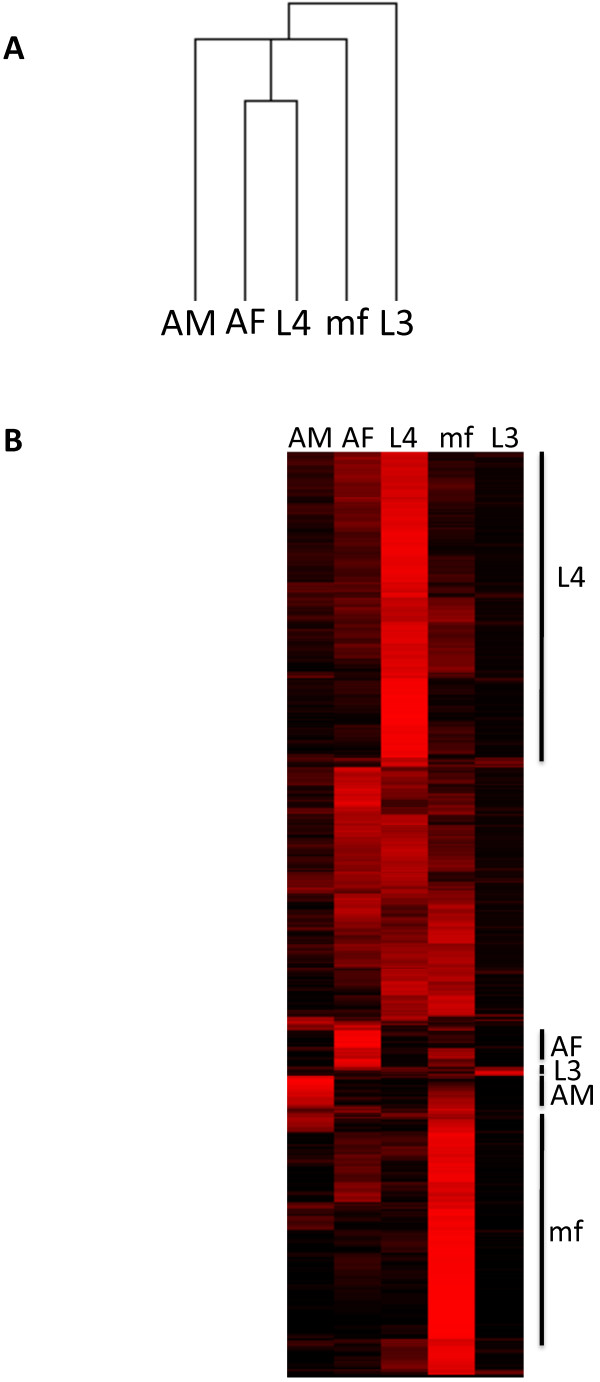
***D*** **.** ***immitis*** **life cycle transcriptome profiles. (A)** Relationships between the various *D. immitis* life cycle stages (AM-Adult Male, AF-Adult Female, mf-microfilariae, L3-3rd stage larva, L4-4th stage larva) as revealed by hierarchical clustering. **(B)** Clustered transcriptomic data of *D. immitis* genes across the various life cycle stages. Only genes expressed in at least one stage are shown (n = 12,819). Each gene is represented by a single row. Data from biological replicates were combined prior to clustering. The color scale ranges from black (no expression) to red (very high expression). Black bars indicate the five clusters expressed predominantly in one *D. immitis* life cycle stage.

The pattern of similarity between *D. immitis* life cycle stages was addressed using hierarchical clustering. The resulting dendrogram (Figure 1A) reveals *D. immitis* AF and L4 larvae had the most similar transcriptional profiles, with AM and mf being more dissimilar and the L3 transcriptional profile was the most distinct (Figure 1A).

As was noted in the previous transcriptomic study of *B. malayi* life cycle stages [38], a large proportion of currently annotated *D. immitis* genes displayed significant transcriptional variability (*i.e.*, were significantly differentially expressed) among five life cycle stages studied herein (Additional file 4: Table S2). To identify groups of *D. immitis* genes preferentially transcribed during specific developmental stages, hierarchical clustering of all *D. immitis* genes was performed (Figure 1B). As indicated by the heatmap, each stage had at least one cluster of genes predominantly expressed in only that stage (Figure 1B). Based on this clustering, nearly 70% of *D. immitis* genes (n = 8717) can be grouped into stage-associated transcriptional patterns (Additional file 5: Table S3) described in further detail below. Transferase activity (GO: 0016740) and glycolipid metabolic processes (GO: 0006664) are the only GO terms enriched among the remaining ~30% of *D. immitis* genes which do not show any stage-associated transcriptional activity.

**Figure 2:**
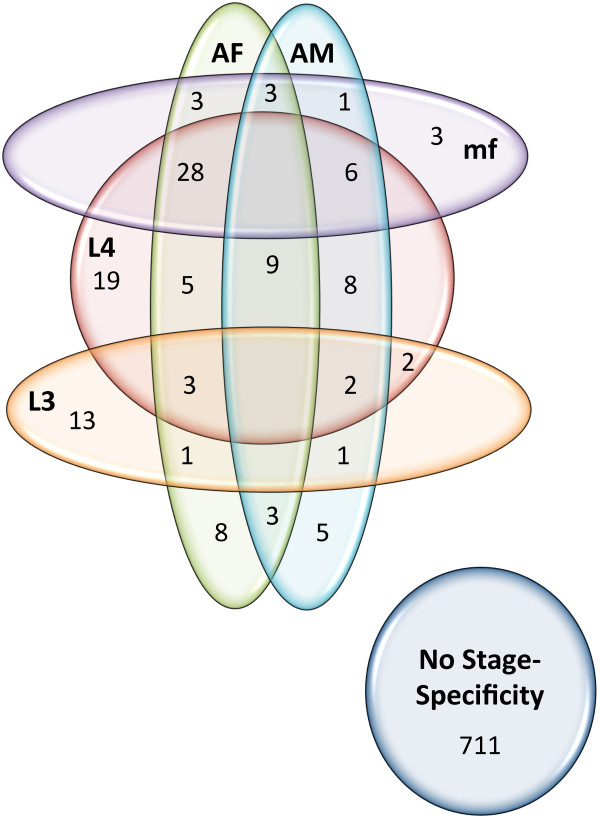
**Venn diagram showing stage-specific expression of the 834 filarial (shared between** ***D*** **.** ***immitis*** **and** ***B*** **.** ***malayi*** **) genes.** Stage-specificity (up to three stages) for each gene was determined for each using differential expression testing by Cuffdiff (Additional file 4: Table S2, q < 0.01). Only life cycle stages expressing the gene (value >0) were considered.

Previous cluster analysis of the predicted *D. immitis* nuclear proteome (version 1.3) with four other nematode proteomes (*B. malayi*, *C. elegans*, *Trichinella sprialis* and *Ascaris suum*) identified 850 ‘filarial-specific’ proteins in clusters, *i.e.*, conserved proteins uniquely shared by *D. immitis* and *B. malayi*, but lacking in the three other nematode species [19]. Comparison of these 850 predicted protein sequences with the improved version 2.2 *D. immitis* proteome (by BLASTp analysis) removed redundancies and yielded 834 predicted proteins which may represent common filarial nematode targets shared between *D. immitis* and *B. malayi*. Assessment of stage-specific transcription of these 834 gene products is listed in Additional file 6: Table S4 and summarized in Figure 2. Notably, only 367 of these genes have functional annotations, twenty of which are listed as hypothetical proteins. Hence, no functional information is available for over half of the 834 genes and many of the existing annotations are relatively vague. Nearly 65% (539) were expressed in all life cycle stages examined while 25 of the 834 *D. immitis* and *B. malayi* specific genes were not expressed in any life cycle stage (Additional file 6: Table S4). The majority of genes (711) displayed no stage-associated transcriptional pattern. Conversely, 123 genes (~15%) were significantly upregulated in at least one life cycle stage (Additional file 6: Table S4, Figure 2). Of these 123 *D. immitis* genes with stage-associated transcriptional patterns, two-thirds were significantly upregulated in the L4 stage of *D. immitis* (alone or in conjunction with other life cycle stages, Figure 2) and include an apoptotic chromatin condensation inducer protein and a number of hypothetical proteins (Additional file 6: Table S4). Seven genes were mutually upregulated between the L3 and L4 stages. Among these 7 L3/L4 upregulated genes are a 227 kDa spindle- and centromere-associated protein and a subunit of a nicotinic acetylcholine receptor. Fifteen additional genes are upregulated in the L3 stage, which may represent potential L3 targets, and include basement membrane proteoglycans and collagen domain containing proteins. Several genes (15) were upregulated in both AM and AF samples (Figure 2). These potential adulticidal targets include genes for adenylate kinase, a c-terminal binding protein and a bzip transcription factor family protein (Additional file 6: Table S4). An additional 48 genes were upregulated in AF (as well as other stages, Figure 2) including two hypothetical proteins, cell death specification protein 2, an ecdysone induced protein and the ecdysone receptor, transcription of which was previously shown to be upregulated in *D. immitis* AF [39]. Upregulation of an additional 23 genes was observed for AM (as well as other stages, Figure 2) including a hypothetical protein and an RNA-binding domain containing protein (Additional file 6: Table S4).

### Gender-associated transcripts

In the context of all five *D. immitis* life cycle stages examined, 414 transcripts and 502 transcripts display AM and AF-associated transcription, respectively (Figure 1B). GO enrichment analysis indicates functional categories such as phosphorylation and DNA repair are overrepresented among the AM associated genes (Additional file 7: Table S5). As expected, structural molecules related to male reproductive structures and sperm generation dominate the male-associated transcripts (Additional file 7: Table S5). These functionalities within *D. immitis* AMs strongly correlate with those previously identified in *B. malayi*[38]: structural molecule activity and proteins associated with protein phosphorylation were prominent among *B. malayi* male associated transcripts.

GO enrichment analysis revealed genes involved in cell division and protein/DNA complexes were overrepresented in *D. immitis* AF, as well as uterus development and chitin binding/processing, likely due to oocyte development (Additional file 7: Table S5). These findings differ from previous transcriptomic studies in *B. malayi* that identified enriched transcription factor activity, nuclear receptor activity, collagens/structural constituents of the cuticle and serpin activity in AF [38]. As described below, we observed enriched serpin transcripts in *D. immitis* mf and cuticle/collagens in *D. immitis* mf, L3 and L4, not adult females. These differences may be attributable to experimental differences (the *B. malayi* life cycle transcriptome is based on only one sample, *i.e.,* no biological replicates were run) or may represent differences between the two species.

A direct pairwise comparison of only AM and AF samples reveals that over 1400 genes display gender-biased transcriptional patterns, the majority (n = 986) of which are upregulated in females. GO enrichment of the remaining 484 male-biased transcripts strongly reiterates the male-associated functional categories observed in the five-way comparison of life cycle stages (Additional file 8: Table S6). Once again, transcripts involved in protein phosphorylation, muscle development and structural molecule activity were prominent among the male-associated transcripts (Additional file 8: Table S6). Given the increase in the number of female-biased transcripts in the pairwise comparison (986 vs. 502), it is interesting that fewer female-associated functional categories are overrepresented in the pairwise comparison with males (Additional file 8: Table S6) than the five-way comparison (Additional file 7: Table S5). The most prevalent over-represented functional categories among female-upregulated genes are those involved in development, including tissue development, neurogenesis and nervous system development (Additional file 8: Table S6). Other interesting female-biased functional categories include cell fate commitment/differentiation and sequence-specific DNA binding (Additional file 8: Table S6). Notably, many of these same gender-associated GO terms were enriched in a microarray gene expression study on adult *B. malayi*[40].

Interestingly, no *w*Di genes were significantly differentially expressed between AM and AFs. This is not entirely surprising since similar transcriptomic studies on *w*Oo identified only 26 differentially expressed genes between AM and AF gonads (FDR of 0.05) [33].

### *D. immitis*larval development

Within the context of gene expression among all five examined life cycle stages, a significant portion of *D. immitis* genes are preferentially transcribed in the L4 stage (4375 transcripts), whereas only 58 transcripts appear to be preferentially transcribed in the L3 stage (Figure 1B). However, genes associated with the extracellular matrix and structural components of the collagen/cuticle are significantly enriched among the L3-associated transcripts, and are likely involved in the L3 to L4 molt (Additional file 7: Table S5).

A number of biological processes are highlighted within the transcripts primarily expressed in the cultured L4 stage. Because larvae more than triple in length during the L4 stage [41], it is unsurprising that genes involved in development, more specifically cellular differentiation, larval development and growth predominate the L4 associated transcripts (Additional file 7: Table S5). Genes involved in reproduction and reproductive development were also significantly enriched within the L4-associated gene set. Additionally, prevalent among L4 transcripts (Additional file 7: Table S5) are genes involved in transcription/translation, muscle cell development and locomotion, cellular components and organization, cellular localization and migration, binding (protein/nucleotide/anion/heterocyclic), transport (endocytosis and secretion), apoptosis, pyrophosphatase activity, and cellular protein complex disassembly. Interestingly, *D. immitis* genes involved in multi-organism processes, or a biological process involving another organism of the same or different species, were enriched among the L4 associated gene set. This functional enrichment could suggest the symbiotic relationship between nematode and *Wolbachia* may be more prominent and potentially more targetable during this life cycle stage or is the result of reproductive development taking place in the L4 stage as the nematode prepares for sexual reproduction.

The L3 to L4 molt is a logical target for vaccine development with the goal of disrupting transmission as the nematode transitions from insect vector to mammalian host. Furthermore, immunization with irradiated L3 larvae from filarial nematodes produces robust and long-lasting immunity [42–45]. Although we were unable to sequence the transcriptome of the L3 to L4 transition directly, direct comparison of the L3 and L4 stages may provide important information regarding transcriptional changes required to undergo this critical transition. Pairwise comparison of the mosquito-derived L3 and cultured L4 transcriptomes indicates that these larval transcriptional profiles are notably different. This is largely in contrast with previous results from *B. malayi* that showed the two larval stages displayed fairly similar transcriptional patterns [38]. Because the *D. immitis* L3 to L4 molt typically occurs faster than with *B. malayi* (3 days versus 8 days)[41, 46], this difference may represent species-specific transcriptional differences between the two filarial nematodes Alternatively, the transcriptional differences observed between L3 and L4 larvae of the two species may be the result of underlying experimental differences: the *B. malayi* L4 sample was derived *in vivo,* while the *D. immitis* L4 were cultured *in vitro*.

Examination of the L3/L4 comparison reveals 1450 significantly differentially expressed genes between L3 and L4 stage, (560 L3 upregulated, 890 L4 upregulated). Enriched GO terms among the L3 upregulated transcripts include genes involved in energy metabolism (including oxidoreductase activity), glycogen/carbohydrate synthesis, transport, muscle development, collagens and structural constituents of the cuticle and larval development (molting) (Additional file 9: Table S7). These results largely concur with the previous findings in *B. malayi*, where transcripts involved in glycogen biosynthesis, oxidoreductase activity, transport, collagen and structural constituents of the cuticle were enriched in the L3 stage [38]. However, the serpin activity and peptidase activity found to be enriched within the *B. malayi* L3 gene set were not identified as enriched functions within the *D. immitis* L3 upregulated gene set.

Functional categories overrepresented within the genes that are upregulated in the L4 stage (as compared to the L3 stage) are primarily involved in transcription and splicing, as well as larval development (Additional file 9: Table S7). These also include reproduction and reproductive tract development, meiosis, protein binding/complexes/folding, endocytosis, nervous system development, as well as cellular component organization, a function also enriched within the L4 associated transcripts in the *B. malayi* transcriptome [38]. Notably, excretory-secretory proteins abundant during the *B. malayi* L3/L4 molt [47], including intermediate filament protein, thioredoxins, glutathione peroxidases, γ-glutamyl transpeptidases, macrophage migratory inhibitory factors and galectins display relatively high expression in the L3 and L4 stages of *D. immitis*.

### Cathepsins and cystatins

Cathepsins (cysteine proteases), more specifically the cathepsin L and Z families, of filarial nematodes are thought to be essential to a variety of biological processes. Deactivation of cathepsins, via either inhibitors or RNAi, drastically delayed or prevented L3 molting in *D. immitis* and *O. volvulus*[48–50]. Furthermore, RNAi knockdown of cathepsin L-like proteases (Bm-cpl-1 and Bm-cpl-5) and cathepsin Z-like protease (Bm-cpz-1) in *B. malayi* decreased AF fecundity [50]. Protein inhibitors of cysteine proteases, or cystatins, are another abundant larval protein family that may serve to control the developmental timing of cathepsin activity and initiation of molting. Due to their essentiality in critical developmental processes and expression patterns, cathepsins and cystatins have long been recognized as potential drug and vaccine targets. Recently, vaccination with a mutated cystatin from *B. malayi* significantly reduced the development of *B. malayi* L3 into adults in gerbils [51]. Moreover, a DNA vaccine with *L. sigmodontis* cysteine protease inhibitor-2 and ALT-1 (abundant larval transcript -1) significantly increased immunogenicity and reduced the number of adult worms and microfilaremia in a mouse model [52].

The *D. immitis* genome annotation contains 10 cathepsin L family members, 2 cathepsin Z family members and 3 cystatin homologues. Overall, the *D. immitis* cathepsin L gene family products are highly expressed in the L3 stage and to a lesser extent in the L4 stage (Additional file 10: Figure S2). Likewise, expression of cystatins appears to correlate with the expression of cathepsin L proteins (highest in the L3 stage). Interestingly, the highest levels of expression for one cathepsin Z family member were observed in mf, suggesting this cathepsin may play a role in molting within the vector host.

### Abundant larval transcripts

Homologous proteins ALT-1 and ALT-2 (abundant larval transcripts -1 and -2) are predominantly expressed in the larval stages of *B. malayi* and were proposed as potential vaccine antigens due to their larval-specificity, high expression levels and lack of a human ortholog [53]. Stage-specific transcriptomic studies in *B. malayi* confirmed the presence of ALT-1 and ALT-2 in both larval stages examined (L3 and L4), however, transcript [38] and protein [47] levels of both were elevated in the L3 stage. The only orthologous protein in *D. immitis*, Di-20/22 L (nDi.2.2.2.g08197), originally identified as an L3 to L4 molt excretory-secretory protein [54], is highly expressed in the L3, L4 and mf stages (no significant difference in expression of Di-20/22 L is observed among any of the larval stages).

### *D. immitis*microfilarial transcriptome

One ultimate objective in filarial research is the development of adulticidal therapies. While not the optimal target, drugs or vaccines that target the mf stage could complement existing treatments (especially in light of recent reduced efficacy reports) and serve as a strategy to limit transmission (particularly in highly endemic areas). In fact, previous studies have proven the feasibility of a vaccine based on mf-derived antigens [55–57]. A large proportion of *D. immitis* transcripts displayed microfilarial expression bias (3368) when compared to the other four stages examined (Figure 1B, Additional file 5: Table S3). Within this group of transcripts, GO terms related to transport are significantly overrepresented (Additional file 7: Table S5). More specifically, the transport of monovalent cations (Na^+^ and K^+^), oxygen and metal ions are overrepresented among mf-associated transcripts. Similar to findings observed in the transcriptomic studies on *B. malayi* microfilariae [38], nucleic acid binding and terms related to transcription are overrepresented among *D. immitis* mf-associated transcripts. Cuticle/collagen formation (procollagen-proline dioxygenase activity) was previously identified as overrepresented within *B. malayi* AF [38]. However, we observe enrichment of cuticle/collagens in the *D. immitis* microfilarial stage.

Interestingly, genes involved in eukaryotic cilium are significantly overrepresented in *D. immitis* mf transcripts (Additional file 7: Table S5). Electron microscopy studies first described ‘unusual’ cilia in the anterior portion of *D. immitis* microfilariae in 1968 [58]. The cilia are not limited to *D. immitis* mf, but are found in other nematode species as well [59]. While unusual at the time, further structural studies indicated these ciliated structures were likely part of modified axon terminals of the amphids and phasmids, chemosensory organs of nematodes found in the anterior and posterior of the animal, respectively [60]. Elevated transcription levels of genes involved in the formation of these ciliated structures as well as the development of synaptic structures (Additional file 7: Table S5) suggests the amphids and/or phasmids are likely either developing or fully developed within the microfilarial stage of *D. immitis* and may therefore play a role in chemosensation and migration behavior.

Gamma-aminobutyric acid (GABA) signaling is another process enriched within mf-associated *D. immitis* transcripts (Additional file 7: Table S5). Interestingly, the effectiveness of macrocyclic lactones such as ivermectin is derived from its agonistic activity of glutamate-gated Cl^-^ channels [61] and GABA_A_ receptors [62, 63]. Mammalian GABAergic neurons are protected from ivermectin by the blood–brain-barrier and therefore relatively unaffected by the drug. However, ivermectin has been shown to cause bodywall and pharyngeal muscle paralysis in nematodes (especially in the larval stages) [61]. It is therefore not surprising that ivermectin administration prior to adulticidal treatment causes a rapid clearing of mf from dogs since *D. immitis* mf appear to contain especially high levels of these GABAergic receptors.

Binding, more specifically tetrapyrrole/heme binding, is functionally overrepresented among *D. immitis* microfilarial transcripts (Additional file 7: Table S5). As heme-containing enzymes (*e.g.*, cytochromes) are often associated with oxygen binding and metabolism, it is not surprising that oxygen binding was also overrepresented in mf-associated transcripts. These findings are specifically interesting due to the proposed symbiotic provisioning of certain metabolites (such as heme and riboflavin) between *Wolbachia* and their obligate nematode hosts (described below) [12].

Another particularly interesting GO term overrepresented among *D. immitis* mf-associated transcripts was DNA integration (GO: 0015074, Additional file 7: Table S5), or the process in which a segment of DNA is incorporated into another, usually larger, DNA molecule such as a chromosome. As might be expected, the transcripts attributed to this GO term enrichment encode for integrases and pao retrotransposon peptidase family proteins. By transposing and propagating within the genome, retrotransposons possess the ability to disrupt essential genes within an organism. Many organisms, including *B. malayi*[64], have been shown to utilize small RNAs in order to counteract the effects of such transposons. However, unlike those in *B. malayi*, all pao type retrotranposons present in the *D. immitis* genome have been fragmented and thus inactivated [19]. Indeed, the *D. immitis* genome was the first sequenced metazoan genome completely lacking any functional transposable elements [19]. In the recent *B. malayi* life cycle transcriptomics study [38], the 4 (Bm1_18655, Bm1_21505, Bm1_36915 and Bm1_57480) of 28 annotated *B. malayi* retrotransposons that were actually transcribed were all transcribed within the microfilarial stage. Thus, the transcription of these inactivated pao retrotransposons in *D. immitis* (especially in the microfilarial stage) likely represents an evolutionary remnant.

Recently, the relationship between filarial nematodes and *Wolbachia* has been of great interest in the study of horizontal or lateral gene transfers (LGTs), where one organism acquires DNA from another organism. Indeed, it appears that widespread LGT has occurred from *Wolbachia* into their arthropod and nematode hosts [65, 66]. Based on the genome sequences, it is estimated that ~24% of the *Wolbachia* genome has been transferred to *D. immitis*[19]. However, only 9 of those candidate LGTs are potentially full-length gene transfers. This appears to differ significantly from *B. malayi* in which it is estimated that only ~15% of the *Wolbachia* genome has been transferred to the nematode, but 36 of these LGTs appear to be full-length [66]. This phenomenon has not yet been studied in the context of the nematode/*Wolbachia* life cycle, but it is interesting to postulate based on our transcriptomic results in *D. immitis* that LGTs may be more likely to occur during the mf stage when enzymes involved in this process (integrases) are highly expressed.

### Chitinase transcription

**Figure 3:**
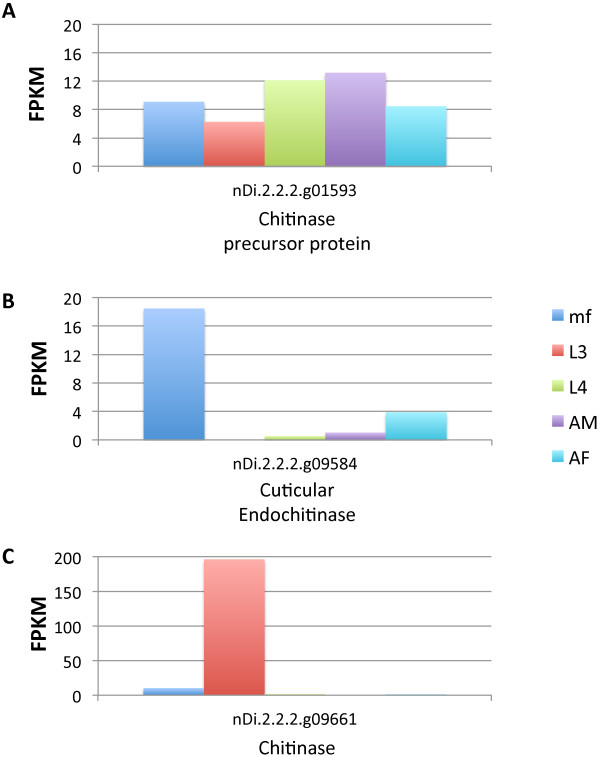
***D. immitis*** **chitinase expression.** Expression profiles (FPKM values) of the *D. immitis* chitinase precursor protein **(A)**, cuticular endochitinase **(B)** and chitinase **(C)** genes.

Many studies have identified microfilarial-specific chitinase activity in filarial nematodes that release sheathed microfilariae (*e.g.*, *B. malayi*, *Wuchereria bancrofti* and *Loa loa*) purportedly involved in exsheathment and/or penetration of the vector midgut [67, 68]. Therefore, unsurprisingly, chitinase activity was previously identified as upregulated in *B. malayi* mf [38]. Conversely, filarial species that shed unsheathed mf, including species such as *D. immitis*, *Acanthocheilonema viteae* and *Onchocerca volvulus*, have been shown to utilize a temperature specific (37°C) L3-specific chitinase, homologous to the *B. malayi* mf-specific chitinase, to possibly degrade the cuticle during the L3 to L4 molt within the mammalian host. Of the three annotated chitinase genes within the *D. immitis* genome, the chitinase precursor protein (nDi.2.2.2.g01593) was consistently transcribed throughout the life cycle (Figure 3A). Although numerous studies have shown a lack of chitinase activity in *D. immitis* mf [67, 68] and specific chitinase activity in the L3 stage [69, 70], we observed significant upregulation of the cuticular endochitinase gene (nDi.2.2.2.g09584) in mf as compared to the L3 stage (Additional file 4: Table S2, Figure 3B). When describing the L3-specific chitinase of *A. viteae*, Wu *et al*., identified a protein of similar molecular weight as the L3-specific chitinase present in mf that displayed the opposite temperature specificity (expression at 27°C, not 37°C) [69], suggesting the presence of an additional chitinase functional within the insect vector. Hence, there is some evidence to support our findings that expression of this endochitinase gene does occur in unsheathed microfilariae of certain nematode species. The exact role of endochitinase in unsheathed mf that do not require chitinases to infect the vector species (*D. immitis* mf do not penetrate the midgut of the insect vector but rather enter the Malpighian tubes through the posterior lumen of the vector midgut [71]), remains unclear. However, chitinase may be important to help the microfilariae migrate through the chitinous peritrophic membrane that surrounds the blood meal. Additionally, because L1 to L3 development for many filarial nematodes within the vector is intracellular, chitinase may be required to invade the host cell. As expected, expression levels of the chitinase gene (nDi.2.2.2.g09661) were much higher in the L3 stage (Figure 3C) than any other chitinase-related gene (nDi.2.2.2.g01593 or nDi.2.2.2.g09584) in any other life cycle stage (note difference in scales between Figure 3A/3B and Figure 3C).

### Transcriptional profiles of the *Wolbachia*endosymbiont (*w*Di)

Hierarchical clustering of transcript expression revealed the *w*Di transcriptional profile of the L3 sample appears to be quite different from the other life cycle stages (Additional file 11: Figure S3A). Interestingly, the resulting heatmap (Additional file 11: Figure S3B) clearly shows comparatively high expression of a number of *w*Di genes during the mf stage in comparison to the other life cycle stages. This may therefore indicate increased transcriptional activity occurs in *Wolbachia* during the mf stage relative to other life cycle stages. Likewise, although very low *w*Di transcriptome coverage was observed in the L4 stage (Table 2), a significant portion of genes appear to be highly transcribed (Additional file 11: Figure S3B), suggesting *Wolbachia* transcription may be substantial during this stage. Although stage-associated gene clusters were identified (Additional file 11: Figure S3B, Additional file 12: Table S8), no functional categories (GO terms) were enriched among any life cycle stage. However, transcripts involved in certain processes were frequently associated with a distinct life cycle stage, *e.g*., in accordance with previous reports suggesting that *Wolbachia* numbers rapidly increase during the L4 stage in filarial nematodes [1, 35–37], a number of critical cell division proteins fall within the L4 cluster (Additional file 12: Table S8). Godel *et al*. [19] suggested a number of *w*Di genes that may be suitable drug targets. The development of drugs that disrupt particular life cycle stages, especially adults and L4s, would be of interest. Specific targets that have been suggested include proteins involved in nucleic acid synthesis (DnaB), enzymes involved in fatty acid synthesis (FabZ and AcpS) [19] and the previously identified anti-*Wolbachia* target, FtsZ, a cell division protein [72]. Our *w*Di transcriptomic data revealed that these potential drug targets all exhibit preferential expression in the mf stage, but also displayed low levels of expression in both adult males and females (Additional file 13: Table S9). Interestingly, expression of only one (DnaB) or two (DnaB and FtsZ) of these potential *Wolbachia* targets was detected for the L3 and L4 stages, respectively.

The *w*Di transcriptomes of mf and L3 were the most significantly different from one another and produced 254 differentially expressed genes, all of which were upregulated in mf. Notably, *w*Di genes including *murF, mraY, murG* and *murJ* (involved in peptidoglycan synthesis), the gene for *hemB* (porphobilinogen synthase, part of the heme biosynthetic pathway), a gene involved in pyrimidine biosynthesis (dihydroorotate dehydrogenase) and *virB9* (one component of the type 4 secretion system) are all upregulated in mf compared to the L3 stage and detailed further below. Among the 107 genes differentially expressed between the L3 and L4 stages, genes involved in peptidoglycan synthesis (*murF*, *murG* and *murJ*), heme biosynthesis (*hemB*), the type 4 secretion system (*virB6* and *virD4*), the Sec translocase (SecF and SecD), and riboflavin biosynthesis *(ribA*) were all upregulated in the L4 stage compared to the L3 stage (Additional file 3: Table S1). Conversely, the heme biosynthesis gene, hemE, and a component of the twin-arginine transporter (*tatA*) were upregulated in the L3 stage compared to the L4 stage (Additional file 3: Table S1). Among the 188 genes differentially expressed between mf and L4 stages were DNA recombination and mismatch repair proteins (RmuC and MutL), *virB11* (type 4 secretion system protein), *yajC* (Sec translocase), *tatA* and *hemE* (Additional file 3: Table S1), all of which were upregulated in the mf stage compared to the L4 stage.

**Figure 4:**
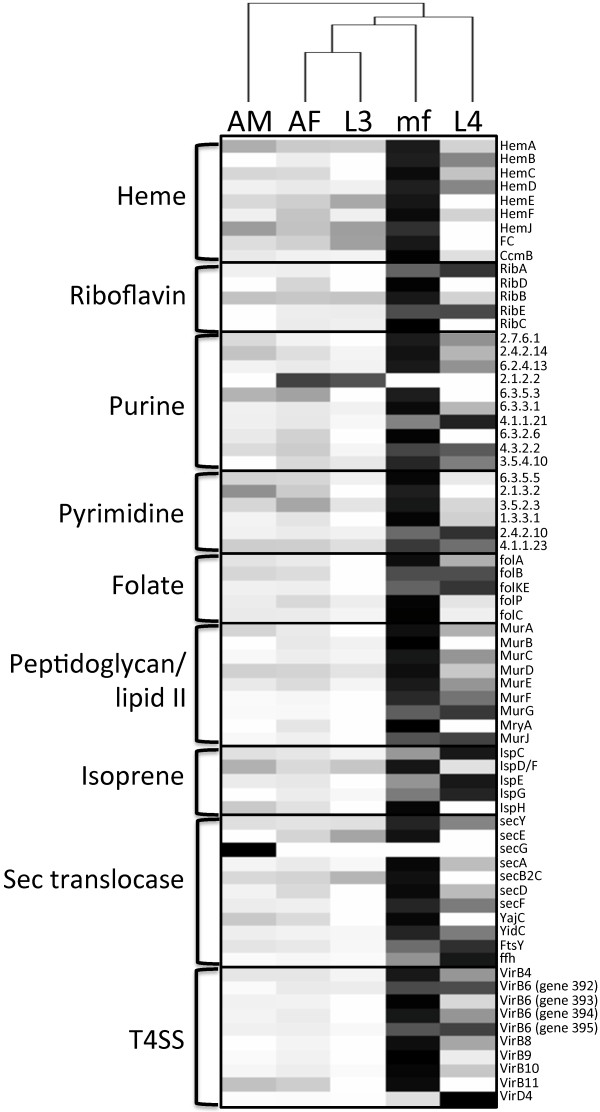
**Expression profiles of** ***Wolbachia*** **metabolic pathways and genes of interest throughout the** ***D*** **.** ***immitis*** **life cycle.** Heatmap showing the expression (normalized FPKM values) of genes involved in the synthesis of heme, riboflavin, purines, pyrimidines, folate, peptidoglycan/lipid II, isoprenoid, as well as components of the Sec translocase and the type IV secretion system. Each gene is represented by a single row. Data from biological replicates were combined prior to clustering. The color scale ranges from white (no expression) to black (very high expression).

While the host-symbiont relationship between filarial nematodes and *Wolbachia* cannot be reduced to one process or factor, a number of obvious relationships can be inferred based on genetic profiling of the two organisms [12, 73, 74]. *D. immitis* lacks specific genes required for *de novo* synthesis of purines, pyrimidines and other cofactors (heme and riboflavin). Similar to *Wolbachia* from *B. malayi* (*w*Bm) [12] these pathways are complete within the *w*Di genome [19]. Conversely, biosynthetic pathways of other vitamins and cofactors such as Coenzyme A, NAD, biotin, lipoic acid, ubiquinone and pyridoxal phosphate are incomplete in *w*Di and thus, may be supplied to the endosymbiont by the host. Although previous transcriptomic studies found little evidence of metabolite provisioning between *Wolbachia* and *O. ochengi*[33], we focused our analysis on transcription of these specific *w*Di pathways (Figure 4, Additional file 13: Table S9), which may provide further information on the evolutionary biology of *Wolbachia* and highlight opportunities for further drug targeting and development.

### Heme synthesis

Serving as an essential cofactor in a number of critical biological processes, heme is a virtually indispensable part of life [75]. The iron-containing tetrapyrrole is synthesized via a highly conserved enzymatic pathway. Similar to *B. malayi*[12, 76], genome sequencing of *D. immitis* and *w*Di revealed that, like other nematodes, *D. immitis* lacked homologues for nearly every enzymatic step in the heme biosynthetic pathway, while *w*Di retained an intact and plausibly functional heme pathway [19]. Although all *D. immitis* life cycle stages display at least low levels of expression for most genes in the pathway, the entire *w*Di heme biosynthesis pathway was predominantly expressed in the mf stage (Figure 4). This is an especially intriguing result given that tetrapyrrole/heme binding was functionally overrepresented in the *D. immitis* mf transcriptome (described above, Additional file 7: Table S5). Moreover, the fact that feedback inhibition does not appear to be affecting transcriptional levels of genes in the beginning of the pathway (*e.g. hemA*) [75, 77], as well as high levels of expression of the CcmB heme exporter in the microfilarial stage (Figure 4) suggest the possibility that heme, while being synthesized in *Wolbachia*, may indeed be exported to the nematode host in a stage-specific manner. The suggestion that heme requirements may be high during the mf stage is particularly interesting given that mf typically survive for long periods of time in the blood of the mammalian host, where heme is seemingly plentiful. However, this stockpiling of heme may be related to a lack of heme availability in the upcoming insect vector stages of parasite development. Recent investigations have found that the capability of *Plasmodium spp* to synthesize heme is critical for parasite development in the mosquito stages [78], suggesting that heme availability may be limiting within the mosquito. A similar scenario may exist for filarial nematode development: microfilariae may be accumulating stores of heme prior to mosquito-induced heme deprivation. The potential for this *Wolbachia-*nematode metabolite provisioning to be stage-specific in filarial worms as our data suggests warrants further investigation.

### Riboflavin synthesis

The synthesis of riboflavin, a precursor to flavin moieties often utilized as enzyme cofactors, is another critical biosynthetic pathway retained in the *w*Di genome and absent in the *D. immitis* genome [12]. Interestingly, the riboflavin synthesis pathway appears complete and functional within *w*Bm and *w*Di, but with the exception of one gene (*ribA*) has been completely pseudogenized in *w*Oo [33]. Again, although transcription of the pathway was evident in nearly every life cycle stage, all genes in the pathway were expressed at relatively high levels in mf stage (Figure 4). RibA and RibE are also highly expressed in the L4 stage, while low levels of transcription for all other genes in the riboflavin pathway were transcribed in the L4 stage (Figure 4). The *w*Di transcriptomic data differ slightly in comparison to previous *w*Bm qRT-PCR data where expression of *ribA* was greatest in the L3 stage and lowest in the mf stage [79].

### Nucleotide synthesis

*De novo* nucleotide synthesis pathways are often lost in endosymbiotic organisms [12], but are maintained in *w*Di [19] and may be used to supplement the host nucleotide pool. Transcripts involved in the *w*Di *de novo* purine biosynthesis pathway are prominently expressed in the mf stage and the L4 stage (Figure 4), with the exception of one gene (phosphoribosylglycinamide formyltransferase, E.C. 2.1.2.2, fig_82301.12.peg.508), for which expression was detected only in AF and L3 samples. All genes in the pyrimidine *de novo* synthesis pathway are highly transcribed in the mf stage and to a lesser extent in the adult stages (Figure 4). Although no transcription of the second step (E.C. 2.1.3.2) in the pyrimidine synthesis pathway is observed for either larval stage (L3 or L4), the final two enzymatic steps within the pathway are highly expressed in the L4 stage (Figure 4). Interestingly, in agreement with previous reports suggesting *Wolbachia* proliferate within the L4 stage of filarial nematodes [1, 35–37], it appears that both nucleotide biosynthesis pathways in *w*Di are highly transcribed in the L4 stage. However, transcription of nucleotide biosynthesis genes is also highly elevated in the mf stage, when relatively little *Wolbachia* replication is occurring [35, 36]. Hence, while transcription of *w*Di nucleotide biosynthesis genes in the L4 stage appears to correlate with *Wolbachia* DNA synthesis, the increased transcription of these nucleotide biosynthesis pathways in the mf stage (when *Wolbachia* numbers remain low within the nematode) suggests stage-specific supplementation of the *D. immitis* nucleotide pool by *Wolbachia*.

### Folate synthesis

Folate synthesis is intricately linked to a number of critical cofactors and metabolites including heme and purine biosynthesis, as well as methylation of DNA [80]. Interestingly, *w*Bm cannot synthesize folate due to a lack of the first two enzymes in the pathway (FolA and FolB) [12]. However, both *w*Di and *w*Oo have retained these genes and can likely synthesize folate [19, 33]. The *w*Di life cycle transcriptome revealed the entire pathway was transcribed in every life cycle stage examined except for the L3 stage. The entire 5-step pathway is apparently highly expressed in mf stage, while the two intermediate steps (FolB and FolKE) were most highly expressed in L4 stage (Figure 4).

### Lipid II synthesis

Because members of the *Wolbachia* genus maintain the genes necessary for lipid II synthesis (*Mur* operon) within their reduced genomes [73, 81], yet lack genes required to synthesize peptidoglycan, the cell wall structure/components may be very different in *Wolbachia* compared to other bacteria. Additionally, recent evidence suggests that this pathway is functional in *Wolbachia*[82] and may play a role in cell division [81], thus making it a worthwhile anti-*Wolbachia* drug target. Once again, based on our transcriptomics data, evidence of complete expression of this *w*Di pathway exists only in AF and mf stages, (Figure 4). Although transcription of certain genes within this pathway is apparently quite high in the L4 stage and to a lesser extent in AMs, no transcripts for *murB* and *mraY* genes were detected in these stages. Interestingly, the enzyme responsible for the formation of lipid I (MraY), was only detected in mf and AFs while MurG, the enzyme responsible for the conversion of lipid I to lipid II, was detected in every stage except for L3. Other genes likely involved in the formation of the pentapeptide moiety of peptidoglycans include *metC* (cystathione beta-lyase, fig_82301.12.peg.810) and *ddl* (D-alanine-D-alanine ligase, fig_82301.12.peg.613). The exact composition of the pentapeptide component of *Wolbachia* peptidoglycan remains unknown, however the presence of *ddl* in the reduced genome strongly suggests the incorporation of D-isomer amino acids. *Wolbachia* were originally thought to lack genes for the racemases necessary to produce D-isomers from L-isomers (typically L-alanine or L-glutamate). However, although classically recognized as a component of the methionine biosynthesis pathway, recombinant MetC from *w*Bm was recently shown to have *in vitro* non-canonical L-alanine racemase activity [83] and likely has a role in peptidoglycan synthesis in *Wolbachia*. Similar to genes in the *Mur* operon, transcript levels of these two genes, *ddl* and *metC*, are highest in the mf and L4 stages. Likewise, transcript levels of the peptidoglycan lipid II flippase, MurJ, thought to be involved in flipping peptidoglycan chains across the membrane into the periplasm, were significantly higher in the L4 and mf stages (Additional file 3: Table S1, Figure 4).

### Isoprenoid synthesis

Isoprenoids are critical metabolites synthesized by all living organisms via two possible pathways: the mevalonic acid (MVA) pathway, utilized by yeast and animals [84, 85] or the methylerythritol 4-phosphate (MEP) pathway present in most bacteria, protozoa and algae [86]. With the exception of plants, most organisms exclusively utilize only one of these pathways for isoprenoid precursor biosynthesis [87]. The genome of *w*Bm revealed the presence of a nearly complete MEP pathway (all but the gene for 1-deoxy-D-xyulose-5-phosphate synthase), for the biosynthesis of isoprenoids [12]. Likewise, *w*Di also lacks the enzyme responsible for the first step in the MEP pathway. As previously suggested for *w*Bm [12], it is possible the missing gene-product (1-deoxy-D-xylulose-5-phosphate) is supplied to *w*Di by *D. immitis*. The MEP pathway of *Wolbachia*, which is absent in humans and nematodes, exemplifies an ideal target for the development of specific anti-*Wolbachia* therapies. Our transcriptomic data suggests that although some genes in the pathway were expressed at considerably higher levels in the L4 than other stages (IspC, IspE and IspG), transcription of other steps in the pathway are extremely low (IspD/F and IspH) (Figure 4). Likewise, expression of various genes in the pathway was completely absent in the L3 (IspE and IspH) and AM stages (IspG). This apparent lack of transcription may simply be the result of the extremely low *w*Di transcriptome coverage reported in some stages (Table 2). Expression of all genes involved in the *w*Di isoprenoid biosynthetic pathway was detected in the AF and mf stages, albeit at a lower frequency than those preferentially expressed in the L4 stage.

### Wolbachia secretion systems

*Wolbachia* protein secretion systems impart a molecular mechanism by which the bacteria may interact with the nematode host. The Sec protein translocation system is responsible for the translocation of unfolded protein across the cell membrane into the periplasmic space. Transmembrane proteins, SecY and SecE, form the core of the protein-translocating channel. A third transmembrane protein, SecG associates with SecYE to form a heterotrimeric complex. Although not required for translocation, the addition of SecG, to form SecYEG, stimulates translocation through the channel at low temperatures (20°C) [88]. SecE, SecY and SecA (the ATPase providing the energy for translocation) have been shown to be absolutely essential to bacterial viability, making the Sec translocase an attractive anti-*Wolbachia* drug target [88, 89]. Interestingly, *w*Di from *D. immitis* L4s only expressed the SecY portion of the transmembrane complex (Figure 4). The non-essential SecG was *only* expressed in *w*Di from AMs. Additionally, SecY is expressed in *w*Di from AM samples, however SecE was not expressed in this stage (Figure 4). With the exception of SecG (which as aforementioned is non-essential and was only expressed in AM), every gene in the *w*Di Sec pathway was transcribed in *D. immitis* mf. This supports a functional Sec translocation system in the mf stage. The driving force for protein translocation via the Sec pathway, SecA, was expressed in every life cycle stage examined while transcripts for SecB, a chaperone that targets proteins to the translocation complex, were detected in every stage except L4 (Figure 4).

An accessory complex (SecDFyajC) comprised of three additional proteins (SecD, SecF and YajC) interacts with SecYEG [88]. Curiously, the exact role of the SecDFyajC complex is not clear. It is not required for protein transport *in vitro* however, *in vivo* protein transport is severely affected by a lack of either SecD or SecF [88]. All three components of the *w*Di SecDFyajC complex were transcribed in AM, AF and mf stages of *D. immitis*, while only the two essential proteins of this complex (SecD and SecF) were expressed in the L4 stage. Interestingly, *Wolbachia* from the third larval stage (L3) of *D. immitis* did not express any part of this complex (SecDFyajC, Figure 4).

Other Sec translocase accessory proteins include: YidC which interacts with Sec translocase and is involved in membrane insertion of both Sec-dependent and Sec-independent proteins [88]; a protein component of the signal recognition particle, ffh, that targets certain unfolded proteins to the membrane for export; and FtsY the proposed receptor for the signal recognition particle [89]. Expression of these three accessory proteins was detected in every *D. immitis* life cycle stage tested, but particularly high in the L4 stage (Figure 4).

The Sec-independent twin arginine translocation (Tat) protein translocation system is found in most bacteria and generally comprised of multiple protein components. While thought to be essential to protein transport via the Tat system [90], *Wolbachia* lack the *tatB* gene, but like other α-proteobacteria likely maintain a functional TatAC translocase [91]. Translational fusion experiments in *E. coli* revealed TatA is the most highly expressed Tat component [92] and expected to be at a 40:1 molar ratio with TatC [90]. Expression of *w*Di *tatA* (fig_82301.12.peg.349) was highest in the L3 stage, less in the AM, AF and mf stages and undetected in the L4 stage. Alternatively, *tatC* (fig_82301.12.peg.851) was highly expressed in mf and L4 stage, less in AM and AF stages and not at all in the L3 stage (Additional file 13: Table S9). The FPKM values for the *D. immitis* AM, AF and mf stages suggest a ~5-24 fold higher expression of *w*Di *tatA* over *tatC*. The observed lack of expression of *w*Di *tatC* in the L3 stage may simply be due to transcript levels being extremely low and beyond the threshold of detection.

Secretion by *Wolbachia* requires translocation not only across the plasma membrane into the periplasm (via the Sec or Tat systems described above), but additionally transport across the outer membrane is required. The multi-subunit type IV secretion system (T4SS) is highly conserved and evolutionarily maintained in *Wolbachia* from insects, arthropods and filarial nematodes [79, 93, 94]. Likely due to an intracellular lifestyle, *Wolbachia* express a minimally functional T4SS lacking four pilus-associated proteins (VirB1, B2, B5 and B7). The remaining eight constituents of the T4SS are clustered within the genome: one cluster comprised of genes encoding VirB3, B4 and B6; the other cluster encodes VirB8, B9, B10, B11 and VirD4 [94, 95]. Similar to *w*Bm [79], the VirB8-VirD4 operon in *w*Di (82301.12.peg.724-728) is preceded by the *ribA* gene (82310.12.peg.723, Figure 4), the first enzyme in riboflavin biosynthesis. Genes for VirB4 and four VirB6 components constitute the other T4SS operon (82301.12.peg.391-395), however, the gene for *virB3* was absent in the most recent annotation of the *w*Di genome (version 2.2). Closer examination reveals *virB3* appeared in the first version of the *w*Di genome (83201.4.peg.46), but the relatively small gene was omitted from the second annotation. Multiple reads from our transcriptome data mapped to this region of the genome (wDi22.scaf1:777977–778268), validating the location and transcription of *virB3* (Additional file 14: Figure S4). All components of the *w*Di T4SS are highly expressed in *D. immitis* mf, and to a lesser extent in AFs (Figure 4). All other life cycle stages (AM, L3 and L4) lacked transcription of one or multiple critical components of the T4SS (Additional file 13: Table S9). Interestingly, *D. immitis* L4s exhibited high transcription levels of all *w*Di T4SS components except for *virB11*, an ATPase required for assembly of the T4SS, for which no expression was detected in this stage. It is noteworthy that the *Wolbachia* Sec translocase, the Tat translocation system and the type IV secretion system were all highly expressed in *D. immitis* mf, especially since the majority of *Wolbachia* proteins previously detected in *B. malayi* excretory-secretory products were of microfilarial origin [47].

One interesting attribute of the T4SS is its ability to mediate not only protein transport, but nucleic acid transfer as well [94, 96]. Possibly such an ability to transport DNA is involved in LGT from *Wolbachia* to the nematode host. Interestingly, in another potential stage-specific host-endosymbiont interaction, genes involved in DNA integration were upregulated in *D. immitis* mf transcripts (Additional file 7: Table S5) while the T4SS appears to be highly transcribed in *Wolbachia* from *D. immitis* mfs. Again, while simply conjecture at this point, it is interesting to postulate that LGTs may be more likely to occur during the mf stage since LGT-promoting transcripts are expressed in both organisms at this stage.

## Conclusions

Stage-specificity of host-symbiont interactions has been demonstrated in entomopathogenic nematodes and their associated enterobacterial symbionts [97], but has been largely unreported in filarial nematodes [33]. Ideally, future therapeutic strategies will target both *D. immitis* and *Wolbachia* and therefore require a greater spatial and temporal understanding of parasite-endosymbiont relationship. This dynamic mixed-transcriptome study adds to the growing body of literature on filarial nematode gene expression and has revealed interesting correlations between *Wolbachia* and *D. immitis* throughout the nematode life cycle. Moreover, this data provides an invaluable resource with which to develop improved therapeutic strategies for *D. immitis* and potentially other closely related filarial nematodes.

### Availability of supporting data

The mapped reads are available in the NCBI short read archive accession SRP048819.

## Electronic supplementary material

Additional file 1: Dataset S1: Transcript FPKM values for *Dirofilaria immitis* life cycle transcriptome. (XLSX 53 KB)

Additional file 2: Figure S1: **(A)** Distribution of *D. immitis* gene densities (FPKM coverage) for each *D. immitis* life cycle stage (biological replicates grouped). Pairwise comparison of *D. immitis* AM **(B)**, AF **(C)**, mf **(D)**, L3 **(E)** and L4 **(F)** biological replicates. Each point represents a single gene. (ZIP 6 MB)

Additional file 3: Table S1: List of significantly (q <0.01) differentially expressed *w*Di genes. (XLSX 872 KB)

Additional file 4: Table S2: List of significantly (q <0.01) differentially expressed *D. immitis* genes. (XLSX 1 MB)

Additional file 5: Table S3: Stage-associated *D. immitis* gene lists. (XLSX 150 KB)

Additional file 6: Table S4: Stage-Specific Expression of the 834 *D. immitis* Genes Uniquely Shared with *B. malayi*. (XLSX 68 KB)

Additional file 7: Table S5: List of over-represented GO terms for stage-associated *D. immitis* genes. (XLSX 69 KB)

Additional file 8: Table S6: List of over-represented GO terms for direct AM and AF pairwise comparison. (XLSX 37 KB)

Additional file 9: Table S7: List of overrepresented GO terms for direct L3 and L4 pairwise comparison. (XLSX 51 KB)

Additional file 10: Figure S2: *D. immitis* cathepsin and cystatin expression. Expression profiles (FPKM values) of cathepsin L and Z family members, as well as cysteine protease inhibitors (cystatins). (PDF 81 KB)

Additional file 11: Figure S3: **(A)** Hierarchical clustering reveals relationships between *Wolbachia* transcription profiles during the various *D. immitis* life cycle stages. **(B)** Clustered transcriptomic data of *w*Di genes across the various life cycle stages. Only genes expressed in at least one stage are shown. Each gene is represented by a single row. Data from biological replicates were combined prior to clustering. The color scale ranges from black (no expression) to red (very high expression). (PDF 42 KB)

Additional file 12: Table S8: Hierarchical clustering of *w*Di expression throughout *D. immitis* life cycle reveals stage-associated (as indicated by X) *w*Di genes. (XLSX 63 KB)

Additional file 13: Table S9: FPKM values for *w*Di transcriptome throughout *D. immitis* life cycle stages. (XLSX 125 KB)

Additional file 14: Figure S4: IGV visualization of transcriptomic reads mapping to the putative location of *w*Di *virB3*. The selected region spans the gap between the *w*Di lysyl-tRNA synthetase gene (82301.12.peg.390) and the *w*Di *virB4* gene (82301.12.peg.391), where the *virB3* gene annotation was omitted from version 2.2 of the *w*Di genome. Reads mapped to this region of the genome (wDi22.scaf1:777977–778268), are indicated (blue bars) for each *D. immitis* biological replicate). (PDF 101 KB)

## Abbreviations

AF: Adult female
ALT: Abundant larval transcript
AM: Adult male
*D. immitis*: *Dirofilaria immitis*
FDR: False discovery rate
FPKM: Fragments Per Kilobase of transcript per Million mapped reads
GABA: Gamma-aminobutyric acid
GO: Gene ontology
IGV: Integrated Genomics Viewer
L3: Third-stage larvae
L4: Fourth-stage larvae
LGT: Lateral gene transfer
mf: microfilariae
MEP: Methylerythritol 4-phosphate
MVA: Mevalonic acid
Tat: Twin arginine translocase
T4SS: Type 4 secretion system
*w*Bm: *Wolbachia* from *Brugia malayi*
*w*Di: *Wolbachia* from *Dirofilaria immitis*
*w*Oo: *Wolbachia* from *Onchocerca ochengi.*
