# Supplementary figures and images for: Concurrent transcriptional profiling of Dirofilaria immitis and its Wolbachia endosymbiont throughout the nematode life cycle reveals coordinated gene expression
Source: BMC Genomics. 2014 Nov 29;15(1):1041. doi: 10.1186/1471-2164-15-1041 (PMC4289336; doi:10.1186/1471-2164-15-1041)

**A**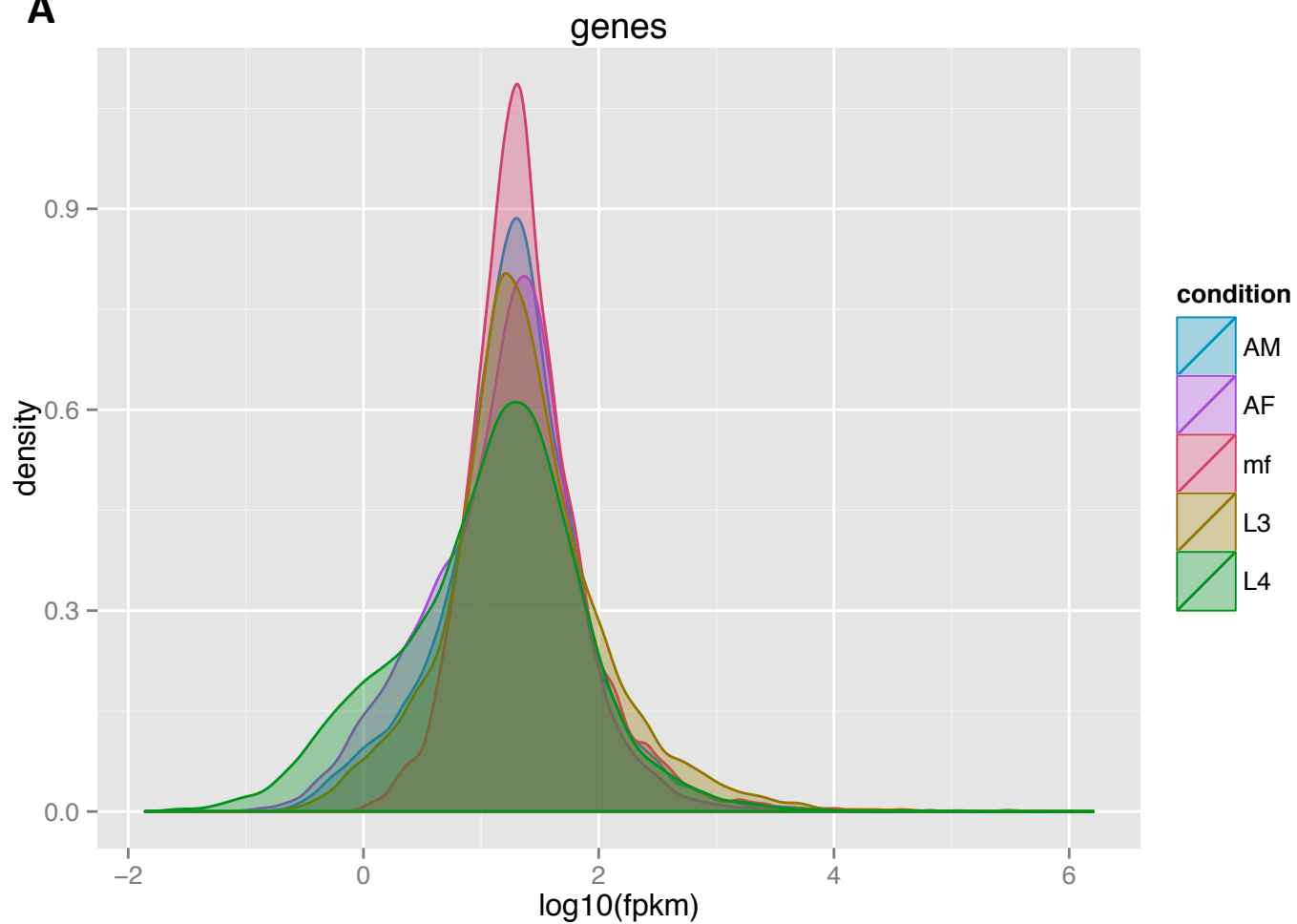

Supplement: Supplementary file 2 — Additional file 2: Figure S1: (A) Distribution of D. immitis gene densities (FPKM coverage) for each D. immitis life cycle stage (biological replicates grouped). Pairwise comparison of D. immitis AM (B), AF (C), mf (D), L3 (E) and L4 (F) biological replicates. Each point represents a single gene. (ZIP 6 MB) [file 12864_2014_6864_MOESM2_ESM.zip › figure S1/1839503231140373_fig5.pdf]

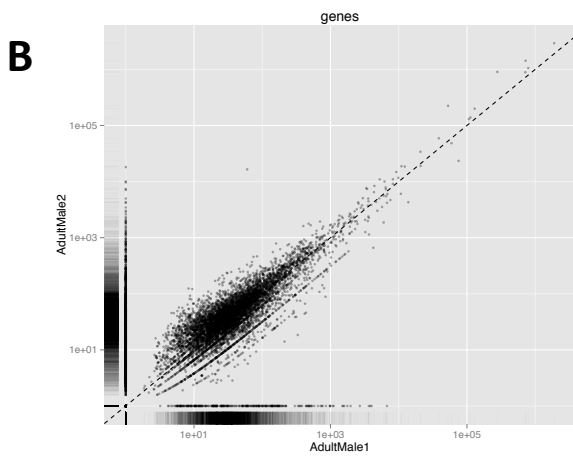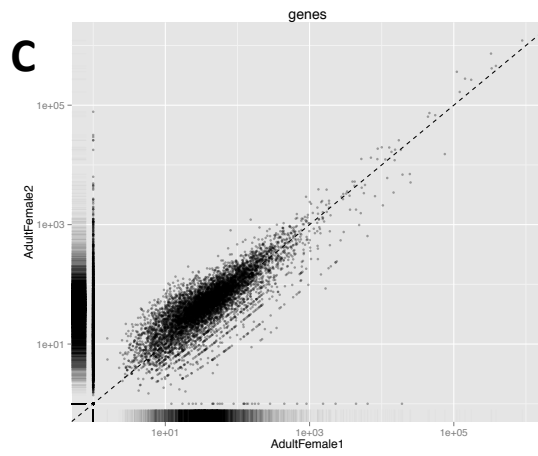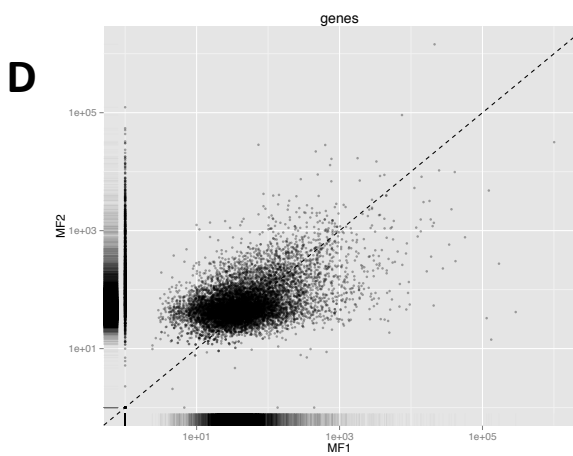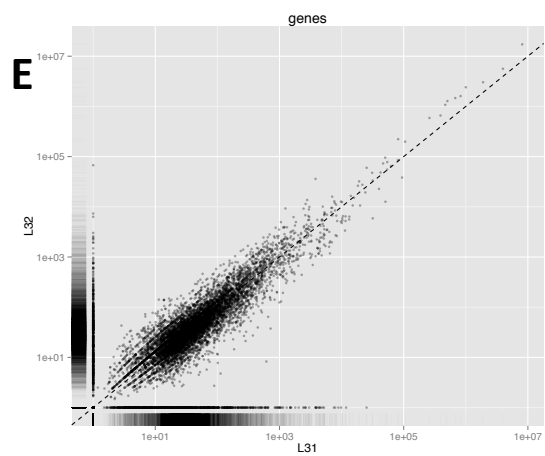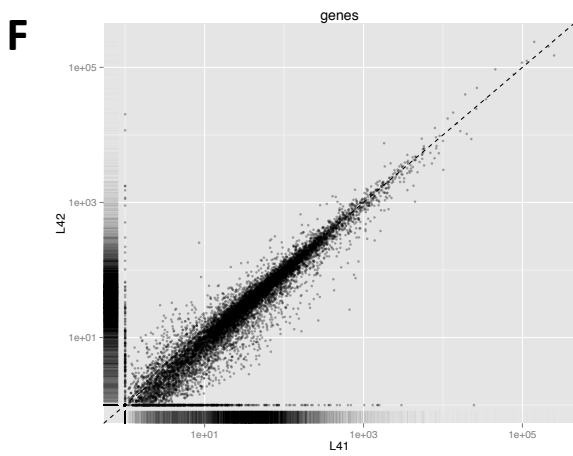

Supplement: Supplementary file 2 — Additional file 2: Figure S1: (A) Distribution of D. immitis gene densities (FPKM coverage) for each D. immitis life cycle stage (biological replicates grouped). Pairwise comparison of D. immitis AM (B), AF (C), mf (D), L3 (E) and L4 (F) biological replicates. Each point represents a single gene. (ZIP 6 MB) [file 12864_2014_6864_MOESM2_ESM.zip › figure S1/1839503231140373_fig9.pdf]

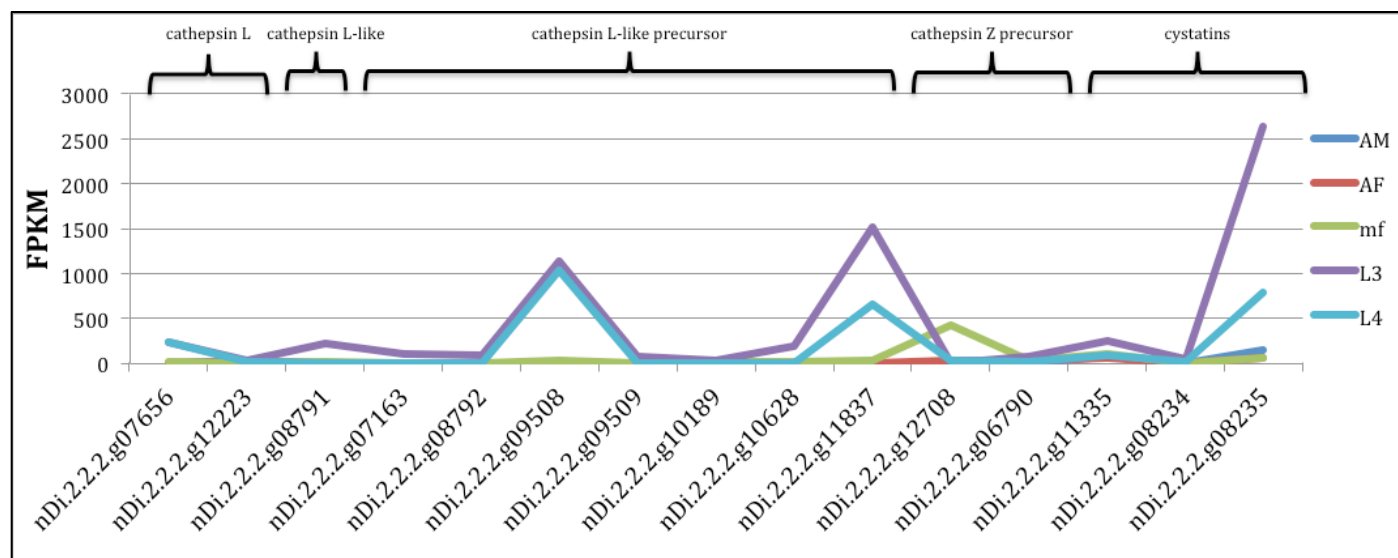

Supplement: Supplementary file 10 — Additional file 10: Figure S2: D. immitis cathepsin and cystatin expression. Expression profiles (FPKM values) of cathepsin L and Z family members, as well as cysteine protease inhibitors (cystatins). (PDF 81 KB) [file 12864_2014_6864_MOESM10_ESM.pdf]

**A**

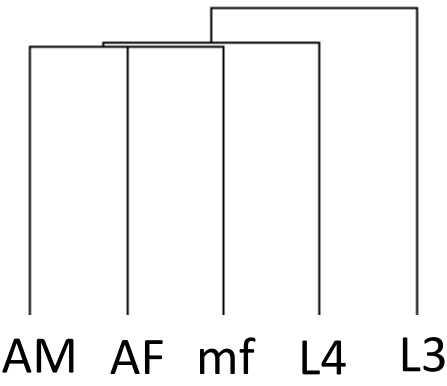

**B**

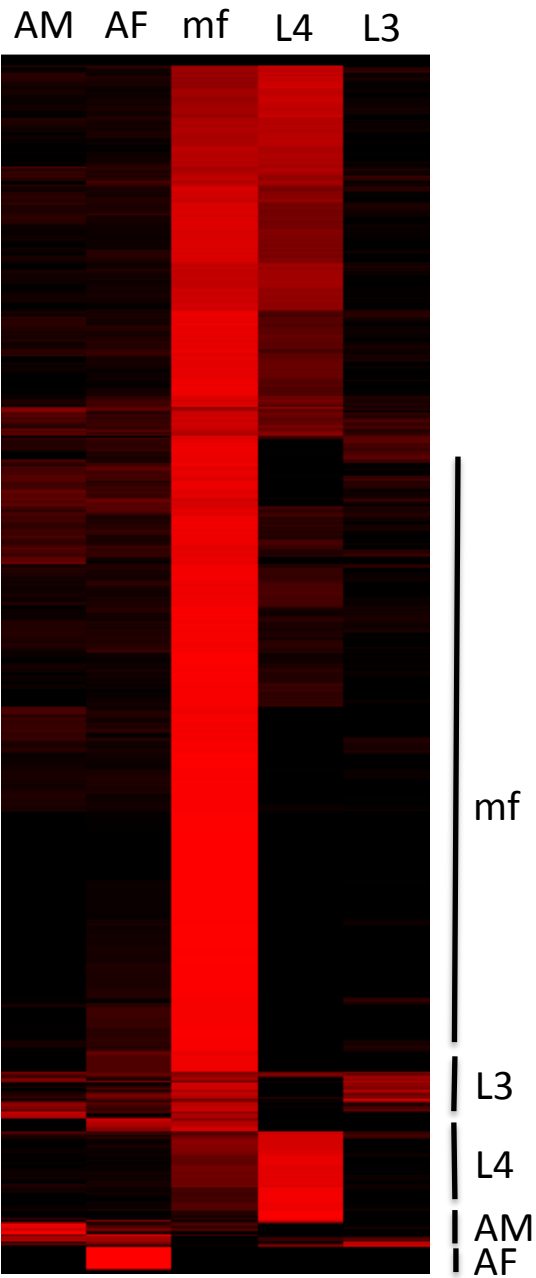

Supplement: Supplementary file 11 — Additional file 11: Figure S3: (A) Hierarchical clustering reveals relationships between Wolbachia transcription profiles during the various D. immitis life cycle stages. (B) Clustered transcriptomic data of wDi genes across the various life cycle stages. Only genes expressed in at least one stage are shown. Each gene is represented by a single row. Data from biological replicates were combined prior to clustering. The color scale ranges from black (no expression) to red (very high expression). (PDF 42 KB) [file 12864_2014_6864_MOESM11_ESM.pdf]

## wDi22.scaf1

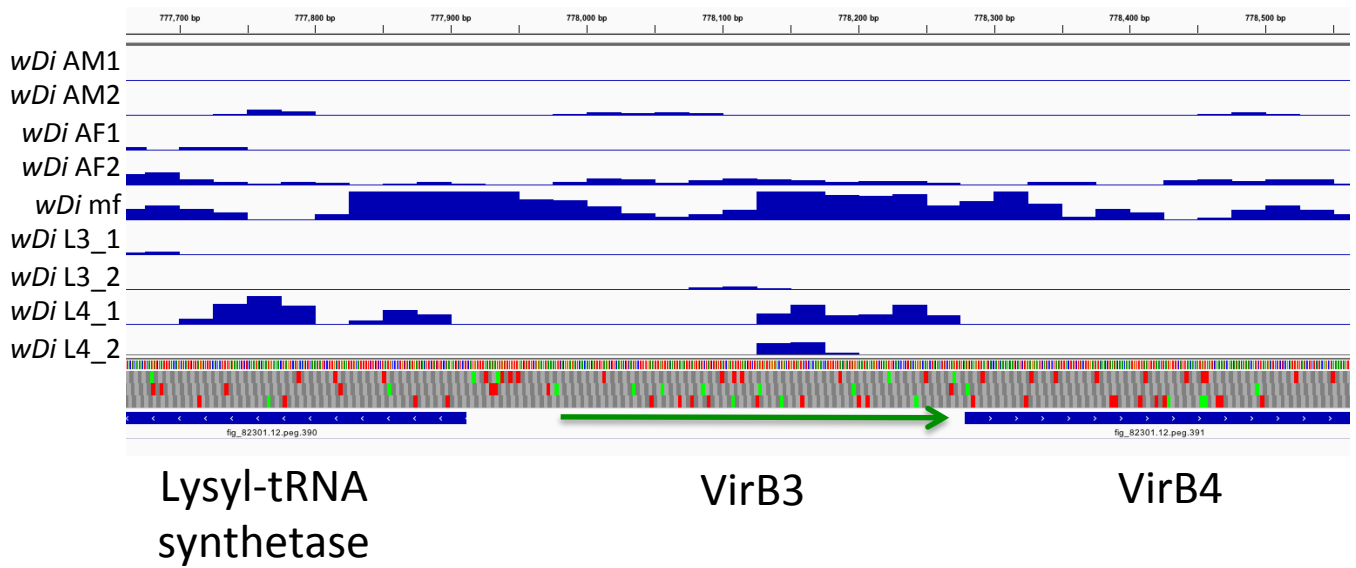

Supplement: Supplementary file 14 — Additional file 14: Figure S4: IGV visualization of transcriptomic reads mapping to the putative location of wDi virB3. The selected region spans the gap between the wDi lysyl-tRNA synthetase gene (82301.12.peg.390) and the wDi virB4 gene (82301.12.peg.391), where the virB3 gene annotation was omitted from version 2.2 of the wDi genome. Reads mapped to this region of the genome (wDi22.scaf1:777977–778268), are indicated (blue bars) for each D. immitis biological replicate). (PDF 101 KB) [file 12864_2014_6864_MOESM14_ESM.pdf]
